# Neurodevelopmental Trajectories and Neurodevelopmental Disorders Following Neonatal Intracranial Hemorrhage: A Long-Term Follow-Up Study and Analysis of Influencing Factors

**DOI:** 10.62641/aep.2298

**Published:** 2026-10-15

**Authors:** Su Lin, Ru Yu, Zhiyong Ding, Zhenlang Lin

**Affiliations:** ^1^Department of Neonatology, The Second Affiliated Hospital and Yuying Children's Hospital of Wenzhou Medical University, 325000 Wenzhou, Zhejiang, China; ^2^Center for Neonatal Follow-up Care and Early Intervention, The Second Affiliated Hospital and Yuying Children's Hospital of Wenzhou Medical University, 325000 Wenzhou, Zhejiang, China

**Keywords:** neonatal intracranial hemorrhage, neurodevelopmental trajectory, neurodevelopmental disorders, long-term follow-up, influencing factors

## Abstract

**Background:**

To investigate the neurodevelopmental trajectories, the incidence of neurodevelopmental disorders, and associated influencing factors in children following neonatal intracranial hemorrhage (ICH), thereby providing a reference for clinical intervention and prognostic improvement.

**Methods:**

A total of 80 neonates diagnosed with ICH and admitted to the Neonatal Intensive Care Unit of the Second Affiliated Hospital and Yuying Children’s Hospital of Wenzhou Medical University were enrolled in this study. All participants completed long-term follow-up assessments over a period of 3–5 years. Baseline clinical data and imaging findings were collected. Neurodevelopmental outcomes were evaluated using the Gesell Developmental Schedules, and neurodevelopmental disorders were identified according to the diagnostic criteria for Brain Damage Syndrome (BDS). The characteristics of neurodevelopmental trajectories, the incidence of neurodevelopmental disorders, and relevant influencing factors were systematically analyzed.

**Results:**

At the conclusion of follow-up, the proportion of children with neurodevelopmental abnormalities (defined as any Developmental Quotient [DQ] <85 on the Gesell Developmental Schedules) was 43.75%, while the incidence of neurodevelopmental disorders (defined according to the diagnostic criteria for BDS) was 38.75%. Gesell Developmental Schedule scores at the 1-, 3-, and 5-year follow-ups showed a gradual increase, with statistically significant differences between time points; the mean total scores were 72.36 ± 5.84, 81.57 ± 6.24, and 86.71 ± 5.93, respectively (F = 113.78, *p* < 0.001). Univariate analysis revealed that Papile grade of hemorrhage (*p* < 0.001), hemorrhage location (*p* < 0.001), birth weight (*p* < 0.001), gestational age (*p* < 0.001), perinatal asphyxia (*p* < 0.001), and concurrent hydrocephalus (*p* = 0.003) were significantly associated with neurodevelopmental disorders. Multivariate logistic regression identified parenchymal hemorrhage (odds ratio [OR] = 4.215, 95% confidence interval [CI]: 1.723–10.312, *p* = 0.002), Papile Grade IV (intraventricular hemorrhage with parenchymal hemorrhage) (OR = 3.826, 95% CI: 1.612–9.083, *p* = 0.003), birth weight < 2500 g (OR = 3.561, 95% CI: 1.458–8.697, *p* = 0.005), and perinatal asphyxia (OR = 3.294, 95% CI: 1.347–8.053, *p* = 0.009) as independent risk factors for the development of neurodevelopmental disorders following neonatal ICH.

**Conclusions:**

Although neurodevelopmental outcomes exhibit gradual improvement following neonatal ICH, a substantial proportion of affected children continue to experience long-term neurodevelopmental abnormalities and disorders. Parenchymal hemorrhage, larger hemorrhage volume, low birth weight, and perinatal asphyxia are identified as key determinants influencing the occurrence of neurodevelopmental disorders. Early intervention strategies targeting these risk factors should be implemented in clinical practice to enhance long-term prognosis.

## Introduction

Neonatal intracranial hemorrhage (ICH) represents a severe perinatal complication. With advances in perinatal medicine and neonatal intensive care technology, the survival rate of affected infants has been significantly improved; however, neurodevelopmental sequelae resulting from ICH have emerged as a major concern affecting long-term quality of life [1]. ICH may induce cerebral hypoxia, elevated intracranial pressure, and neuronal injury, subsequently leading to remote outcomes such as motor deficits, cognitive impairments, and epilepsy. A subset of affected children may even manifest learning difficulties and social adjustment disorders during school age [2]. Therefore, elucidating the evolutionary patterns of neurodevelopmental trajectories following ICH and identifying key factors that influence the occurrence of neurodevelopmental disorders are of paramount importance for the formulation of individualized intervention strategies.

The prognosis of ICH is highly variable. Neurodevelopmental outcomes depend mainly on hemorrhage location, hemorrhage volume, associated complications, and the timing of intervention [3]. Infants with mild hemorrhage generally exhibit favorable prognoses; the hemorrhagic foci may be gradually reabsorbed, and their neurodevelopment is not significantly different from that of age-matched peers. In contrast, severe hemorrhage is frequently complicated by hydrocephalus and periventricular leukomalacia, leading to irreversible impairments in motor and cognitive functions [4]. Recent study has further indicated that asymptomatic subdural hemorrhage may be self-limiting, as no statistically significant differences in neurodevelopmental scores were observed between affected infants and healthy controls at the 2-year follow-up assessment [5]. Nevertheless, for symptomatic ICH, systematic long-term follow-up evaluation is essential to ascertain its enduring impact, particularly since severe sequelae such as cerebral palsy and intellectual disability may be delayed in onset until infancy or even school age [6]. Regarding influencing factors, studies have demonstrated that the incidence of remote sequelae following germinal matrix-intraventricular hemorrhage in preterm infants remains considerable, with a substantial proportion of affected children experiencing long-term neurodevelopmental impairments, among which periventricular white matter injury may result in spastic diplegia and visual-cognitive integration deficits [7]. Moreover, the timing of therapeutic intervention is critical for neural repair. The initial 72 hours post-hemorrhage constitute a pivotal window for controlling intracranial pressure and maintaining cerebral perfusion. According to Park *et al*. [8], aggressive and early surgical intervention for intraventricular hemorrhage contributes to better control of intracranial pressure and reduces infection rates and brain damage resulting from delayed shunt insertion. However, comparative studies examining the effects of distinct therapeutic modalities on long-term cognitive function remain relatively scarce. Specifically, differences between neurosurgical intervention and conservative management in promoting hemorrhage resolution and improving prognosis warrant further validation.

The present study was designed to analyze the stage-specific characteristics of neurodevelopmental trajectories within a long-term follow-up cohort of neonates with ICH, to identify independent risk factors for the development of neurodevelopmental disorders, and to provide an evidence base for the early identification of high-risk populations and the optimization of rehabilitative interventions.

## Materials and Methods

### Study Population

A total of 80 neonates diagnosed with ICH who were admitted to the Neonatal Intensive Care Unit of the Second Affiliated Hospital and Yuying Children’s Hospital of Wenzhou Medical University between January 2017 and December 2020 were enrolled in this study.

The inclusion criteria were as follows: (1) fulfillment of the diagnostic criteria for neonatal ICH [9], confirmed by cranial computed tomography (CT) or magnetic resonance imaging (MRI); (2) admission within 24 hours of birth; (3) availability of core clinical data (including demographic characteristics, perinatal history, and imaging findings). The exclusion criteria were as follows: (1) presence of congenital cerebral malformations, genetic disorders, or severe infectious diseases; (2) loss to follow-up, mortality during the follow-up period, or voluntary withdrawal from the study; and (3) concurrent diagnosis of other neurological disorders.

During the initial screening period, a total of 146 neonates with suspected or confirmed ICH were identified. Of these, 66 cases were excluded from the final analysis. The reasons for exclusion were categorized as follows: (1) Failure to meet the inclusion criteria (n = 16): including 16 cases with incomplete clinical data. (2) Meeting the exclusion criteria (n = 50): including 18 cases with congenital cerebral malformations or genetic disorders, 12 cases with severe infectious diseases, 8 cases diagnosed with other neurological disorders, 9 cases lost to follow-up, and 3 cases with withdrawal of informed consent. Thus, a total of 80 participants were included in the final analysis (as shown in Fig. 1). All core demographic, clinical, and outcome data for these 80 participants were complete, with no missing values. The handling of missing data and participant exclusion is explicitly described here and is not repeated in subsequent sections.

**Fig. 1.**
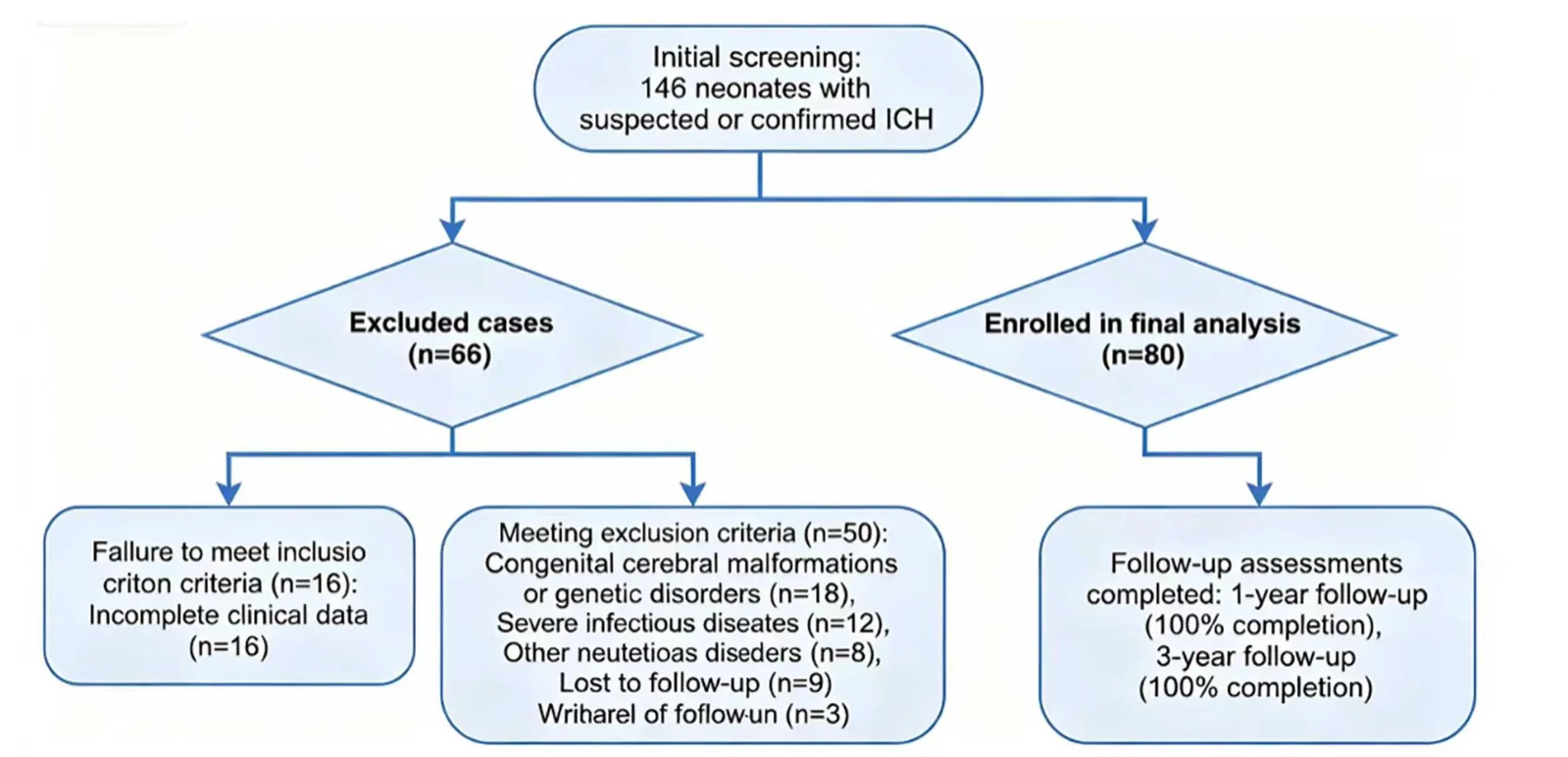
**Flow diagram of participant enrollment, exclusion, and follow-up attrition**.

### Follow-Up Protocol

All enrolled neonates underwent long‑term follow‑up assessments over 3–5 years. Follow‑up evaluations were performed through outpatient clinic visits and telephone interviews.

(1) Growth and physical development indicators: At each visit, body weight, recumbent length, head circumference, and chest circumference were measured using standardized instruments. Age‑ and sex‑specific z‑scores were calculated according to the World Health Organization (WHO) Child Growth Standards [10] and thenational growth standards for children under 7 years [11, 12].

(2) Clinical symptom assessment: A structured questionnaire was administered to guardians to document feeding difficulties, sleep disturbances, abnormal muscle tone, seizure‑like episodes, visual impairment, and hearing impairment. The questionnaire was based on standardized neurodevelopmental follow‑up protocols for high‑risk infants [13, 14]. All reported symptoms were verified by a pediatric neurologist.

(3) Physical and neurological examination: A standardized physical and neurological examination was performed by a pediatric neurologist at each visit, including general physical examination, cranial nerve function, muscle tone (modified Ashworth scale), deep tendon reflexes, primitive reflexes (during the first 6 months), postural reflexes, and gross/fine motor function. Examination procedures followed standardized neonatal neurological assessment protocols [15, 16].

(4) Neurodevelopmental assessment: Neurodevelopmental status was evaluated using the Gesell Developmental Schedules at the 1-, 3-, and 5-year time points. The standardized Chinese revision of the Gesell Schedules [17, 18] was used, and results were expressed as Developmental Quotients (DQs).

(5) Neuroimaging evaluation: Cranial CT or MRI was repeated at the 1-year follow-up and annually thereafter, or earlier if clinically indicated, to assess hemorrhage resolution, secondary brain injury, and structural abnormalities.

### Assessment Measures

#### Neurodevelopmental Assessment

Neurodevelopmental status was evaluated using the Gesell Developmental Schedules [18], which assess five domains: gross motor function, fine motor function, language, personal-social behavior, and adaptive behavior. The scoring criteria were applied in accordance with the established literature [19], wherein the results were expressed as DQs. A DQ of ≥85 was classified as normal development, whereas a DQ of <85 was indicative of developmental delay. Mean scores were calculated for each domain, as well as an overall mean score.

#### Assessment of Neurodevelopmental Disorders

According to the diagnostic criteria of Brain Damage Syndrome (BDS), neurodevelopmental disorders were identified [20], which correspond to Diagnostic and Statistical Manual of Mental Disorders, Fifth Edition (DSM-5)/ International Classification of Diseases, 11th Revision (ICD-11) diagnoses including Global Developmental Delay, Specific Developmental Disorder, Epilepsy, and Cerebral Palsy. Participants were classified as having a neurodevelopmental disorder if they met any one of the following criteria:

(1) Abnormal findings on the 36-item neurological examination: During the period from 1 to 12 months of age, ≥3 positive items, or any one of the following three findings (epilepsy, failure of visual tracking, or absence of orienting response to a rattle), was considered indicative of a neurodevelopmental disorder [20, 21].

(2) Abnormal Gesell assessment: either a global DQ ≤85, or a domain-specific DQ ≤70 in any single functional area [20].

(3) Concurrent abnormalities on neurological examination and developmental scale: one positive item on the 36-item neurological examination in conjunction with either a global DQ ≤90 or a domain-specific DQ ≤80 on the Gesell Schedules [20].

(4) Abnormal Neonatal Behavioral Neurological Assessment (NBNA): The NBNA scale comprises 20 items with a maximum total score of 40; a score ≤35 is considered indicative of an elevated risk for neurological impairment [14, 22, 23].

### Statistical Analysis

Statistical analyses were performed using SPSS software (version 26.0; IBM Corp., Armonk, NY, USA). Continuous variables are presented as mean ± standard deviation ($\bar{x}$ ± s). Normality of continuous data was assessed using the Kolmogorov–Smirnov test (for the total sample of n = 80, with a sample size ≥50). For comparisons among independent groups (e.g., the three treatment groups at baseline), one-way analysis of variance (ANOVA) was used for normally distributed data, with post-hoc pairwise comparisons performed using the least significant difference (LSD) *t*‑test. For categorical variables, frequencies and percentages [n (%)] were compared using the chi-square (χ^2^) test. For repeated measurements of the same cohort at three time points (1-, 3-, and 5-year follow‑ups), one-way repeated‑measures ANOVA was applied, followed by post‑hoc pairwise comparisons using the LSD *t*‑test. Multivariate analysis was performed using logistic regression to identify independent risk factors. A two-tailed *p* value of <0.05 was considered statistically significant.

Regarding missing data: no missing values were present for the core demographic, clinical, or outcome variables among the 80 included participants. A total of 9 participants were lost to follow-up during the study period (as shown in Fig. 1); these participants were excluded from the final analysis, and no imputation was performed. All 80 participants completed the full 3‑ to 5‑year follow-up schedule with complete Gesell assessments at the 1‑, 3‑, and 5‑year time points; thus, there were no incomplete follow-up assessments within the analytic cohort. The handling of missing data (complete‑case analysis) is explicitly noted.

## Results

### Baseline Characteristics of the Study Population

The baseline characteristics of the 80 enrolled neonates with ICH are summarized in Table 1 (Ref. [24]). Among the cohort, 42 (52.50%) were male and 38 (47.50%) were female. Mean birth weight was 2943.63 ± 452.80 g, with 23 cases (28.75%) classified as low birth weight (<2500 g). Mean gestational age was 37.45 ± 1.62 weeks, with 27 cases (33.75%) born preterm (<37 weeks). According to the Papile grading system, 26 cases (32.50%) were classified as Grade I (subependymal/germinal matrix hemorrhage), 21 (26.25%) as Grade II (intraventricular hemorrhage without ventricular dilation), 16 (20.00%) as Grade III (intraventricular hemorrhage with ventricular dilation), and 17 (21.25%) as Grade IV (intraventricular hemorrhage with parenchymal hemorrhage). Perinatal asphyxia was documented in 19 cases (23.75%), and concurrent hydrocephalus was noted in 8 cases (10.00%) (Table 1, Ref. [24]).

**Table 1.** **Baseline data of 80 cases of newborn ICH**.

Characteristic		n (%)
Sex		
	Male	42 (52.50)
	Female	38 (47.50)
Birth weight (g)		
	<2500 g	23 (28.75)
	≥2500 g	57 (71.25)
Gestational age (weeks)		
	<37 weeks	27 (33.75)
	≥37 weeks	53 (66.25)
Papile grade of hemorrhage		
	Grade I (subependymal/germinal matrix hemorrhage)	26 (32.50)
	Grade II (intraventricular hemorrhage without ventricular dilation)	21 (26.25)
	Grade III (intraventricular hemorrhage with ventricular dilation)	16 (20.00)
	Grade IV (intraventricular hemorrhage with parenchymal hemorrhage)	17 (21.25)
Hemorrhage location		
	Parenchymal hemorrhage	28 (35.00)
	Subarachnoid hemorrhage	32 (40.00)
	Intraventricular hemorrhage	15 (18.75)
	Mixed-type hemorrhage	5 (6.25)
Perinatal asphyxia		
	Yes	19 (23.75)
	No	61 (76.25)
Concurrent hydrocephalus		
	Yes	8 (10.00)
	No	72 (90.00)

Note: ICH, intracranial hemorrhage. Papile grade was determined based on cranial imaging findings: Grade I = subependymal/germinal matrix hemorrhage; Grade II = intraventricular hemorrhage without ventricular dilation; Grade III = intraventricular hemorrhage with ventricular dilation; Grade IV = intraventricular hemorrhage with parenchymal hemorrhage [24].

### Longitudinal Changes in Neurodevelopmental Scores Across Follow-Up Time Points

A progressive increase in the mean total Gesell Developmental Schedule score was observed across the follow-up assessments conducted at 1, 3, and 5 years. The 1‑year and 3‑year assessments were completed by all 80 participants, whereas the 5‑year assessment was completed by 71 participants (88.75%). Statistically significant differences were identified in both total scores and domain-specific scores across the follow-up time points (all *p* < 0.001). Notably, scores across all five developmental domains demonstrated gradual improvement over the extended follow-up period, with the adaptive behavior domain exhibiting a relatively pronounced magnitude of improvement (Table 2). At the final follow-up assessment (3–5 years), the proportion of children with neurodevelopmental abnormalities, defined as any DQ <85 on the Gesell Developmental Schedules, was 43.75% (35 out of 80). Given the absence of a healthy control group, the observed increase in scores should be interpreted as within‑cohort changes over time rather than direct evidence of “recovery” relative to normative developmental trajectories. The term “neurodevelopmental trajectory” is used descriptively to denote longitudinal changes in Gesell scores within the study cohort.

**Table 2.** **Comparison of Gesell Development Scale scores at different follow-up time points**.

Follow-up time	n	Gross motor	Fine motor	Language	Personal-social	Adaptive behavior	Total mean score
Follow-up for 1 year	80	70.15 ± 6.38	68.87 ± 5.96	71.24 ± 6.15	73.54 ± 5.68	75.93 ± 5.07	72.36 ± 5.84
Follow-up for 3 years	80	79.81 ± 6.14	77.95 ± 5.80	80.73 ± 5.93	81.33 ± 5.15	85.81 ± 5.72	81.57 ± 6.24
Follow-up for 5 years	71	83.53 ± 5.80	83.74 ± 5.73	85.83 ± 5.80	87.95 ± 5.04	92.12 ± 5.63	86.71 ± 5.93
F		98.56	127.34	119.87	142.65	171.23	113.78
*p*		<0.001	<0.001	<0.001	<0.001	<0.001	<0.001

Note: Data are presented as mean ± SD. At the 5‑year follow‑up, 71 participants completed the assessment; the 1‑year and 3‑year assessments were completed by all 80 participants. Abbreviations: SD, standard deviation; F, F‑statistic from one‑way repeated‑measures ANOVA; *p*, probability value.

At the 5‑year follow‑up, 71 participants completed the assessment (88.75%); the remaining 9 participants (11.25%) had follow‑up durations between 3 and 4.8 years and did not reach the 5‑year time point. Thus, the sample size at 5 years is 71, while the 1‑year and 3‑year assessments were completed by all 80 participants. The population composition across time points was consistent with respect to sex, birth weight, and hemorrhage severity (all *p* > 0.05 comparing the 71 participants with the full cohort).

Post‑hoc pairwise comparisons using the LSD *t*‑test revealed that the total Gesell score at 3 years (81.57 ± 6.24) was significantly higher than at 1 year (72.36 ± 5.84) (mean difference = 9.21, 95% CI: 7.12–11.30, *p* < 0.001). The score at 5 years (86.71 ± 5.93) was significantly higher than at both 1 year (mean difference = 14.35, 95% CI: 12.05–16.65, *p* < 0.001) and 3 years (mean difference = 5.14, 95% CI: 3.21–7.07, *p* < 0.001). Similar patterns were observed for all five domain‑specific scores (all *p* < 0.001).

### Incidence of Neurodevelopmental Disorders and Univariate Analysis

At the conclusion of the follow-up period, neurodevelopmental disorders were identified in 31 of the 80 enrolled neonates, yielding an incidence rate of 38.75%. Among the affected cases, 18 were classified as mild (58.06%), 10 as moderate (32.26%), and 3 as severe (9.68%) (percentages calulated using the 31 diagnosed children as the denominator). Univariate analysis revealed that Papile grade of hemorrhage (χ^2^ = 14.635, *p* = 0.002), hemorrhage location (χ^2^ = 19.400, *p* < 0.001), birth weight (χ^2^ = 12.915, *p* < 0.001), gestational age (χ^2^ = 13.382, *p* < 0.001), perinatal asphyxia (χ^2^ = 16.964, *p* < 0.001), and concurrent hydrocephalus (χ^2^ = 8.901, *p* = 0.003) were significantly associated with the occurrence of neurodevelopmental disorders. In contrast, no statistically significant association was observed between sex and the development of neurodevelopmental disorders (*p* > 0.05) (Table 3).

**Table 3.** **Univariate analysis of the occurrence of neurodevelopmental disorders after ICH in neonates [n (%)]**.

Factor		Normal cognitive function	Neurodevelopmental disorder	χ^2^ value	*p* value
		(n = 49)	(n = 31)			
Sex				0.016	0.899
	Male	26 (53.06)	16 (51.61)		
	Female	23 (46.94)	15 (48.39)		
Birth weight				12.915	<0.001
	<2500 g	7 (14.29)	16 (51.61)		
	≥2500 g	42 (85.71)	15 (48.39)		
Gestational age				13.382	<0.001
	<37 weeks	9 (18.37)	18 (58.06)		
	≥37 weeks	40 (81.63)	13 (41.94)		
Papile grade of hemorrhage				14.635	0.002
	Grade I	21 (42.86)	5 (16.13)		
	Grade II	14 (28.57)	7 (22.58)		
	Grade III	10 (20.41)	6 (19.35)		
	Grade IV	4 (8.16)	13 (41.94)		
Hemorrhage location				19.400	<0.001
	Parenchymal hemorrhage	8 (16.33)	20 (64.52)		
	Subarachnoid hemorrhage	25 (51.02)	7 (22.58)		
	Intraventricular hemorrhage	12 (24.49)	3 (9.68)		
	Mixed-type hemorrhage	4 (8.16)	1 (3.23)		
Perinatal asphyxia				16.964	<0.001
	Yes	4 (8.16)	15 (48.39)		
	No	45 (91.84)	16 (51.61)		
Concurrent hydrocephalus				8.901	0.003
	Yes	1 (2.04)	7 (22.58)		
	No	48 (97.96)	24 (77.42)		

Note: Data are presented as n (%). Abbreviations: χ^2^, chi‑square statistic; *p*, probability value; ICH, intracranial hemorrhage.

### Multivariate Logistic Regression Analysis of Factors Associated With Neurodevelopmental Disorders

Variables demonstrating statistical significance (*p* < 0.05) in the univariate analysis—specifically, hemorrhage location, Papile grade of hemorrhage, birth weight, gestational age, perinatal asphyxia, and concurrent hydrocephalus—were entered as independent variables into a multivariate logistic regression model, with the presence of neurodevelopmental disorders (1 = present, 0 = absent) designated as the dependent variable.

The analysis identified the following factors as independent risk factors for the development of neurodevelopmental disorders following neonatal ICH: parenchymal hemorrhage (OR = 4.215, 95% CI: 1.723–10.312, *p* = 0.002) , Papile Grade IV (intraventricular hemorrhage with parenchymal hemorrhage) (OR = 3.826, 95% CI: 1.612–9.083, *p* = 0.003), birth weight <2500 g (OR = 3.561, 95% CI: 1.458–8.697, *p* = 0.005), and perinatal asphyxia (OR = 3.294, 95% CI: 1.347–8.053, *p* = 0.009). Detailed results are presented in Table 4.

**Table 4.** **Multiple factor Logistic regression analysis of neurodevelopmental disorders after neonatal ICH**.

Variable	Assignment	OR	95% CI	χ ^2^	*p* value
Hemorrhage location					
(reference: subarachnoid hemorrhage)					
Parenchymal hemorrhage	1, others 0	4.215	1.723–10.312	9.874	0.002
Intraventricular hemorrhage	1, others 0	2.156	0.812–5.723	2.135	0.144
Mixed‑type hemorrhage	1, others 0	1.872	0.598–5.862	1.256	0.262
Papile Grade IV	1, others 0	3.826	1.612–9.083	8.624	0.003
(intraventricular hemorrhage					
with parenchymal hemorrhage)					
Birth weight (<2500 g vs. ≥2500 g)	<2500 g = 1, ≥2500 g = 0	3.561	1.458–8.697	7.653	0.005
Perinatal asphyxia (yes vs. no)	Yes = 1, No = 0	3.294	1.347–8.053	6.782	0.009

Note: OR, odds ratio; CI, confidence interval; χ^2^, chi‑square statistic; *p*, probability value; mL, milliliter; g, gram; ICH, intracranial hemorrhage.

### Growth, Clinical Symptoms, and Physical Examination Findings

At the final follow‑up assessment (5 years or last visit), the mean body weight z‑score was –0.42 ± 0.89, mean recumbent length z‑score was –0.38 ± 0.76, and mean head circumference z‑score was –0.51 ± 0.68.

Regarding clinical symptoms reported by guardians during the follow‑up period, the most common symptoms were: feeding difficulties (poor sucking or choking during feeding) in 23 participants (28.75%) during the first 6 months of life, which resolved spontaneously in most cases by 12 months of age; sleep disturbances (excessive crying or difficulty falling asleep) in 31 participants (38.75%); and abnormal muscle tone (hypertonia or hypotonia observed by guardians) in 18 participants (22.50%). Seizure‑like episodes (staring or limb jerking) were reported in 7 participants (8.75%).

Physical examination performed by pediatric neurologists at the 5‑year follow‑up revealed abnormal neurological findings in 23 of the 71 participants who completed the 5‑year assessment (32.39%) . The most frequent abnormalities were: increased or decreased deep tendon reflexes (n = 13, 18.31%), abnormal muscle tone (n = 10, 14.08%), persistence of primitive reflexes beyond 6 months of age (n = 8, 11.27%), and delayed achievement of postural reflexes (n = 6, 8.45%). Cranial nerve examination was normal in all participants. Motor function assessment at 5 years showed that 62 of the 71 participants (87.32%) achieved independent walking; among the remaining 9 participants, 6 (8.45%) walked with assistance and 3 (4.23%) were non‑ambulatory.

## Discussion

This longitudinal cohort study, which involved long-term follow-up of 80 neonates with ICH over a period of 3–5 years, yielded several key findings. First, neurodevelopmental outcomes exhibited gradual improvement over time, with the mean total Gesell Developmental Schedule score increasing from 72.36 ± 5.84 at the 1-year follow-up to 86.71 ± 5.93 at the 5-year follow-up; nevertheless, neurodevelopmental abnormalities (any DQ < 85) persisted in 43.75% of the cohort. Second, the overall incidence of neurodevelopmental disorders reached 38.75%, with mild disorders constituting the predominant subtype. Third, multivariate analysis confirmed that parenchymal hemorrhage, Papile Grade IV hemorrhage, birth weight <2500 g, and perinatal asphyxia are independent risk factors for the development of neurodevelopmental disorders. These findings provide a critical evidence base for the formulation of targeted clinical intervention strategies.

A trajectory of gradual neurodevelopmental improvement was evident, as both the total Gesell scores and the scores across the five specific domains (gross motor, fine motor, language, personal-social, and adaptive behavior) demonstrated consistent increases at the 1-, 3-, and 5-year follow-up assessments. The most notable improvement was observed in the adaptive behavior domain. Two factors may explain this improvement. First, the neonatal brain has considerable neuroplasticity. Unaffected neural regions can compensate for impaired functions through synaptic remodeling and neural stem cell proliferation/differentiation. Second, external interventions (e.g., nutritional support and early rehabilitation training) likely create a more favorable neurodevelopmental environment [25]. Epidemiological studies have shown that GM-IVH is associated with increased risks of cerebral palsy and motor/cognitive delay, with severe IVH conferring even greater susceptibility to these adverse outcomes [26, 27]. Zhou *et al*. [26] conducted a meta‑analysis systematically evaluating neurodevelopmental outcomes in preterm or low birth weight infants with germinal matrix‑intraventricular hemorrhage. Their findings demonstrated that neurodevelopmental outcomes differed significantly across hemorrhage grades, with severe hemorrhage (Grades III–IV) being significantly associated with poorer cognitive, motor, and language outcomes, further supporting the robust association between ICH severity and neurodevelopmental impairment. In comparison to that study, the present investigation extends the follow-up duration to 5 years and provides a more granular analysis of domain-specific developmental disparities, thereby elucidating the rapid improvement characteristic of the adaptive behavior domain. This observation underscores the potential value of prioritizing early cultivation of adaptive skills in clinical rehabilitation protocols.

The incidence of neurodevelopmental disorders at the conclusion of follow-up was determined to be 38.75% (comprising 18 mild, 10 moderate, and 3 severe cases), indicating a substantial long-term risk of cognitive impairment subsequent to neonatal ICH. The pathogenesis of neurodevelopmental disorders involves direct disruption of cognition-related brain regions (e.g., hippocampus and frontal cortex) by hemorrhage. This leads to neuronal necrosis and axonal disruption, compromising information processing and storage. Additionally, hemoglobin degradation products trigger oxidative stress cascades, and inflammatory cytokines infiltrate cerebral tissue, exacerbating brain injury and impairing orientation and memory [28]. Notably, the follow-up period in the present study spanned 3–5 years, encompassing critical windows of childhood cognitive development and thereby providing a more comprehensive representation of long-term neurocognitive trajectories. In contrast to prior investigations with shorter follow-up durations (frequently <2 years), which may fail to capture later dynamic cognitive changes, the results of this study offer a more reliable reference for long-term prognostic evaluation.

Parenchymal hemorrhage was identified as the foremost independent risk factor for neurodevelopmental disorders (OR = 3.872), a finding closely linked to the functional anatomy of the cerebral parenchyma. As the brain parenchyma constitutes a densely populated region of neurons housing neural pathways essential for core cognitive and motor functions, hemorrhage in this area directly inflicts irreversible neuronal injury. Moreover, the mass effect of the hematoma compresses adjacent cerebral tissue, leading to focal circulatory disturbance and exacerbating hypoxic-ischemic injury, which collectively impedes subsequent cognitive development [29]. Cizmeci *et al*. [30] demonstrated that intraparenchymal hemorrhage occurring subsequent to ventricular reservoir taps in neonates with hydrocephalus was significantly correlated with adverse neurodevelopmental outcomes at 2 years of age, further validating the deleterious impact of parenchymal involvement. Compared with that study, the present investigation focused on a general cohort of neonates with ICH, thereby excluding confounding factors associated with specific procedural interventions. This approach clarifies the independent risk attributable to parenchymal hemorrhage per se and provides a quantitative estimate of the associated risk magnitude, offering a more precise metric for the early identification of high-risk infants.

The risk of neurodevelopmental disorders was significantly elevated in neonates with Papile Grade IV hemorrhage (intraventricular hemorrhage with parenchymal hemorrhage) (OR = 3.826). The underlying mechanism is presumed to involve a marked elevation in intracranial pressure associated with larger hemorrhage volumes, which exerts compressive effects on brain tissue and precipitates widespread ischemia and hypoxia. Additionally, the liberation of substantial blood components triggers a systemic inflammatory response, and the accumulation of inflammatory mediators within cerebral tissue accelerates neuronal apoptosis [31]. Although Davis *et al*. [32] reported a positive correlation between hemorrhage volume and adverse neurodevelopmental outcomes in extremely preterm infants with severe ICH, a specific volumetric threshold was not delineated in that study. In the present study, Papile Grade IV hemorrhage, defined as intraventricular hemorrhage with parenchymal extension, was associated with a 3.826‑fold higher risk of neurodevelopmental disorders compared to other grades (OR = 3.826, 95% CI: 1.612–9.083), providing a clinically applicable imaging‑based criterion for risk stratification. This quantitative criterion provides a concrete reference for clinical severity stratification and the development of interventional strategies, facilitating the prompt identification and management of high-risk cases.

Birth weight <2500 g was confirmed as a significant independent risk factor for neurodevelopmental disorders (OR = 3.561). This association may be attributed to the cerebral immaturity characteristic of low-birth-weight infants, particularly those born preterm. Such infants often exhibit incomplete integrity of the blood-brain barrier and fragile cerebral vasculature, which impair neurorepair capacity following hemorrhage. Moreover, low birth weight is frequently accompanied by intrauterine growth restriction and depleted nutritional reserves, factors that further compromise neuronal proliferation and differentiation [33]. Winkelhorst *et al*. [34] similarly documented a strong correlation between low birth weight and adverse long-term neurodevelopment in cases of ICH secondary to fetal and neonatal alloimmune thrombocytopenia. The present study validates this risk factor in a broader population of neonates with ICH, independent of specific etiologic conditions, thereby enhancing the generalizability of the findings. Furthermore, the detailed documentation of the dose-response relationship between birth weight and cognitive function provides a foundation for individualized interventions targeting this vulnerable subgroup.

Perinatal asphyxia was found to confer an increased risk of neurodevelopmental disorders (OR = 3.294). The proposed mechanism involves direct hypoxic-ischemic cerebral insult induced by asphyxia, which precipitates a disruption of energy metabolism and promotes neuronal apoptosis due to insufficient adenosine triphosphate (ATP) production. Simultaneously, asphyxia impairs vascular endothelial integrity, thereby exacerbating the increased vascular permeability associated with ICH. This interaction establishes a deleterious cycle of hemorrhage and hypoxia, resulting in a more extensive zone of cerebral injury [35]. Lakatos *et al*. [36] investigated the neurodevelopmental effect of ICH in asphyxiated neonates treated with hypothermia. While hypoxic-ischemic encephalopathy (HIE)-related MRI abnormalities were significant prognostic factors, ICH per se did not have an independent effect on neurodevelopmental outcomes after accounting for the degree of HIE. The present analysis isolated the independent contribution of perinatal asphyxia, demonstrating that even in the absence of overt hypoxic-ischemic encephalopathy, asphyxia itself significantly elevates the risk of neurodevelopmental disorders. This finding underscores the clinical imperative to implement more intensive intervention protocols—including early optimization of cerebral perfusion and oxygenation—for ICH-affected neonates with a documented history of perinatal asphyxia, with the aim of mitigating secondary brain injury.

## Conclusions

This long‑term follow‑up study of neonates with ICH demonstrated that although neurodevelopmental outcomes gradually improve over time, a substantial proportion of affected children continue to experience neurodevelopmental abnormalities and disorders. Parenchymal hemorrhage, Papile Grade IV hemorrhage, low birth weight, and perinatal asphyxia were identified as independent risk factors for neurodevelopmental disorders. These findings highlight the need for early risk stratification and targeted intervention. Future prospective multicenter studies with larger sample sizes and inclusion of healthy control groups are warranted to validate these results and to further elucidate the causal mechanisms linking hemorrhage characteristics to specific neurodevelopmental outcomes. Additionally, research evaluating the effectiveness of early rehabilitation programs in high‑risk subgroups is needed to guide clinical practice.

## Availability of Data and Materials

The datasets used and analysed during the current study were available from the corresponding author on reasonable request.
